# Comprehensive Characterization of *Escherichia coli* O104:H4 Isolated from Patients in the Netherlands

**DOI:** 10.3389/fmicb.2015.01348

**Published:** 2015-12-02

**Authors:** Mithila Ferdous, Kai Zhou, Richard F. de Boer, Alexander W. Friedrich, Anna M. D. Kooistra-Smid, John W. A. Rossen

**Affiliations:** ^1^Department of Medical Microbiology, University Medical Center Groningen, University of GroningenGroningen, Netherlands; ^2^State Key Laboratory for Diagnosis and Treatment of Infectious Diseases, The First Affiliated Hospital, College of Medicine, Zhejiang UniversityHangzhou, China; ^3^Collaborative Innovation Center for Diagnosis and Treatment of Infectious DiseasesHangzhou, China; ^4^Department of Medical Microbiology, Certe-Laboratory for Infectious DiseasesGroningen, Netherlands

**Keywords:** Shiga toxin-producing *E. coli* –STEC, enterohemorrhagic *E. coli* –EHEC, hemolytic uremic syndrome, outbreak, antimicrobial resistance, whole genome sequencing, phylogenetic analysis

## Abstract

In 2011, a Shiga toxin-producing Enteroaggregative *Escherichia coli* (EAEC Stx2a+) O104:H4 strain caused a serious outbreak of acute gastroenteritis and hemolytic-uremic syndrome (HUS) in Germany. In 2013, *E. coli* O104:H4 isolates were obtained from a patient with HUS and her friend showing only gastrointestinal complaints. The antimicrobial resistance and virulence profiles of these isolates together with three EAEC Stx2a+ O104:H4 isolates from 2011 were determined and compared. Whole-genome sequencing (WGS) was performed for detailed characterization and to determine genetic relationship of the isolates. Four additional genomes of EAEC Stx2a+ O104:H4 isolates of 2009 and 2011 available on NCBI were included in the virulence and phylogenetic analysis. All *E. coli* O104:H4 isolates tested were positive for *stx*2a, *aatA*, and *terD* but were negative for *escV.* All, except one 2011 isolate, were positive for *agg*R and were therefore considered EAEC. The EAEC Stx2a+ O104:H4 isolates of 2013 belonged to sequence type (ST) ST678 as the 2011 isolates and showed slightly different resistance and virulence patterns compared to the 2011 isolates. Core-genome phylogenetic analysis showed that the isolates of 2013 formed a separate cluster from the isolates of 2011 and 2009 by 27 and 20 different alleles, respectively. In addition, only a one-allele difference was found between the isolate of the HUS-patient and that of her friend. Our study shows that EAEC Stx2a+ O104:H4 strains highly similar to the 2011 outbreak clone in their core genome are still circulating necessitating proper surveillance to prevent further outbreaks with these potentially pathogenic strains. In addition, WGS not only provided a detailed characterization of the isolates but its high discriminatory power also enabled us to discriminate the 2013 isolates from the isolates of 2009 and 2011 expediting the use of WGS in public health services to rapidly apply proper infection control strategies.

## Introduction

Shiga toxin-producing *Escherichia coli* (STEC) are a pathogenic group of *Escherichia coli* (*E. coli*) producing the potent cytotoxin Shiga-toxin (Stx) similar to the one produced by *Shigella dysenteriae* serotype 1 (Gyles, 2007). STEC may cause a broad spectrum of illness, ranging from diarrhea to the potentially fatal hemolytic uremic syndrome (HUS) (Farrokh et al., 2013). A subset of STEC can cause bloody diarrhea in humans and they are known as enterohemorrhagic *E. coli* (EHEC) while a subset of EHEC can cause HUS and are known as HUS-associated *E. coli* HUSEC (Mellmann et al., 2008). In addition to Shiga toxin-converting bacteriophages, STEC may contain several other mobile genetic elements encoding virulence factors as pathogenicity islands (PAI), and a large, approximately 90 kb plasmid (pO157; Karch, 2001). Stx production is common to all HUS-associated *E. coli* isolates regardless of their serotype. When the toxin enters the blood stream it binds to receptors on endothelial cells abundantly present in kidneys and brain, leading to neurological sequel and/or to microvascular disease that may result in HUS (Welinder-Olsson and Kaijser, 2005).

In 2011, a large outbreak was reported in Germany caused by an Enteroaggregative *E. coli* (EAEC) O104:H4 strain lysogenized with the Stx2a bacteriophage and thereby becoming an EAEC/STEC hybrid strain (Bielaszewska et al., 2011; Brzuszkiewicz et al., 2011). Besides the *stx2a* gene, this unusual strain had virulence properties of EAEC including plasmid pAA carrying the aggregative adherence fimbriae (AAF) variant I encoded by the *aggA* gene whose expression is regulated by the *aggR* gene. In addition, it contained a protein-coat secretion system (Aat), dispersin (Aap), a putative type VI secretion system (Aai), and a rare combination of serine protease autotransporters of Enterobacteriaceae (SPATEs) genes, i.e., *sepA*, *sigA*, and *pic* (Rasko et al., 2011; Scheutz et al., 2011). It also contained a *terD* gene (tellurite resistance gene as a marker for the *ter* cluster) and a plasmid-borne extended spectrum beta-lactamase (ESBL) gene *bla*_CTX-M-15_ resulting in resistance to several antibiotics (Brzuszkiewicz et al., 2011; Rasko et al., 2011). However, the outbreak clone did not possess the *escV* gene encoding the predicted outer membrane protein and a marker for the locus of enterocyte effacement (LEE) PAI (Müller et al., 2009; Scheutz et al., 2011).

In clinical microbiology, whole genome sequencing (WGS) has already shown its value in outbreak investigations and epidemiological typing due to its high-resolution discriminatory power and detailed virulence profiling, thereby becoming more and more important in routine diagnostics (Mellmann et al., 2011; Rohde et al., 2011; Grad et al., 2012). In this study, we characterized EAEC Stx2a+ O104:H4 strains isolated from a HUS patient and her friend who traveled together to Turkey in 2013 prior to diagnosing the patient with HUS. For this, a WGS approach in parallel with routine phenotypic and genotypic laboratory methods was used. Analyses were performed to get more insight into the antibiotic resistance and virulence profiles of the isolates and to reveal their genetic relationship with the 2011 German outbreak EAEC Stx2a+ O104:H4 isolates.

## Materials and Methods

### *E. coli* Isolates used in This Study

In July 2013, four *E. coli* isolates were obtained from a HUS patient (isolate 338) and her friend (isolates 381-1, 381-3, and 381-4). They were compared to three EAEC Stx2a+ O104:H4 strains named 7N, 8G, and LB227103 which were a kind gift of Dr. Alexander Mellmann (Institute of Hygiene, University of Muenster, Muenster, Germany) and were isolated during the 2011 German outbreak period from stool samples of patients, submitted to the National Consulting Laboratory for HUS in Münster, Germany, between May 23 and June 2, 2011 (Bielaszewska et al., 2011; Pritchard et al., 2012). In addition, publically available genomes of five previously reported strains (TY-2482, 2011C-3494, 2009EL-2050, 2009EL-2071, and 55989) were included in virulence and phylogenetic analyses. Detailed information on the isolates used in this study is shown in **Table 1**.

**Table 1:** Characteristics of isolates analyzed in this study.

Isolate ID	Date of isolation	Country of isolation	Clinical manifestations	*stx* subtype	Serotype	Extended spectrum beta-lactamase (ESBL)	Multilocus sequence typing (MLST)	Source
338	July 2013	Netherlands	HUS	2a	O104:H4	Negative	ST-678	This study
381-1	July 2013	Netherlands	Diarrhea	2a	O104:H4	Positive	ST-678	This study
381-3	July 2013	Netherlands	Diarrhea	NA^a^	O126:H2	Positive	ST-10	This study
381-4	July 2013	Netherlands	Diarrhea	2a	O104:H4	Negative	ST-678	This study
7N	2011	Germany	Unknown	2a	O104:H4	Positive	ST-678	This study
8G	2011	Germany	Unknown	2a	O104:H4	Positive	ST-678	This study
LB227103	2011	Germany	HUS	2a	O104:H4	Positive	ST-678	Bielaszewska et al., 2011; Pritchard et al., 2012
TY-2482	2011	Germany	HUS	2a	O104:H4	Positive	ST-678	Rohde et al., 2011
2011C-3493	2011	USA	HUS	2a	O104:H4	Positive	ST-678	Ahmed et al., 2012
2009EL–2050	2009	Republic of Georgia	Bloody diarrhea	2a	O104:H4	Negative	ST-678	Ahmed et al., 2012
2009EL–2071	2009	Republic of Georgia	Bloody diarrhea	2a	O104:H4	Negative	ST-678	Ahmed et al., 2012
55989	1995	Central African Republic	Diarrhea	NA^a^	O104:H4	Negative	ST-678	Touchon et al., 2009; Miko et al., 2013


*^a^NA = Not applicable as these isolates are *stx* negative.*

### Diagnostic Procedures

Fecal samples from the HUS patient (patient 338) and her friend (patient 381) were collected for diagnostic purposes at Certe Laboratory for Infectious Diseases as described previously (de Boer et al., 2015). Shortly, fecal samples were screened for the presence of the virulence genes *stx*1, *stx*2, and *escV* by real-time PCR (qPCR) and *stx*-positive samples were subsequently enriched in Brilliant green bile (BGB) broth. After DNA isolation from the enriched broth, qPCR was performed for detection of the EAEC pAA plasmid encoding genes *aggR* and *aatA*, and O-serogroup encoding genes (O26, O91, O103, O104, O111, O121, O145, and O157). In parallel, part of the BGB broth was plated on CHROMagar STEC and Sorbitol MacConkey agar plates (Mediaproducts BV, Groningen, the Netherlands). To obtain a pure isolate from the selective agar plates, more than 30 colonies of each patient were analyzed for the presence of *stx* genes by qPCR and for ESBL production by using CHROM ID ESBL agar (bioMérieux, Marcy I’Etoile, France). In addition, sorbitol fermentation and tellurite resistance of the isolates were determined by observing their ability to grow on CT-SMAC plates (Sorbitol MacConkey agar with Cefixime and Tellurite). In addition, conventional PCRs for *stx-*subtypes 2a, 2b, 2c, 2d, 2e, 2f, and 2g were performed to determine the subtype of the *stx*2 gene as described previously (Scheutz et al., 2012). Finally, a multiplex PCR targeting typical molecular features of the 2011 outbreak strain, i.e., the presence of the *wzy*_O104_ (O antigen polymerase as a marker for the *E. coli* O104 gene cluster), *fliCH4* (encoding the H4 specific flagellin), *stx2*, and *terD* genes was performed (Bielaszewska et al., 2011; Zhang et al., 2012).

### Antimicrobial Resistance

Antibiotic resistance patterns of the four isolates of 2013 (338, 381-1, 381-3, and 381-4) and three isolates of 2011 (7N, 8G, and LB227103) were analyzed using VITEK2 (bioMerieux, Marcy l’Etoile, France) and E-Test (bioMérieux, Marcy l’Etoile, France) following EUCAST guidelines. Presence of ESBL, carbapenemases and AmpCs genes was confirmed using the Check-MDR CT103 assay according to the manufacturer’s protocol (Check-Points BV Wageningen, the Netherlands).

### DNA Isolation

For repetitive sequence based PCR fingerprinting and WGS, genomic DNA was extracted by the UltraClean^®^ microbial DNA isolation kit (MO BIO Laboratories, Carlsbad, CA, USA) according to the manufacturer’s protocol. For other molecular assays DNA was extracted using a DNeasy blood and tissue kit (QIAGEN, GmbH, Hilden, Germany) following the manufacturer’s protocol.

### Multilocus Sequence Typing (MLST)

Multilocus sequence typing was performed using a 3130xl Genetic Analyzer (Applied Biosystems) to sequence internal fragments of seven housekeeping genes (*adk*, *fumC*, *gyrB*, *icd*, *mdh*, *purA*, and *recA*) according to the protocol described in the *E. coli* MLST databases (http://mlst.warwick.ac.uk/mlst/dbs/Ecoli/documents/primersColi_html). The alleles and sequence types (ST) were assigned in accordance with the same database.

### Virulence Properties

To assess the presence of known *E. coli* virulence genes, a DNA microarray analysis was performed using the *E. coli* genotyping combined assay kit according to the manufacturer’s protocol (Clondiag, Alere Technologies, GmbH, Jena, Germany; Geue et al., 2010).

### Repetitive Sequence based PCR (rep-PCR)

Isolated DNA was amplified using the DiversiLab *Escherichia* kit for repetitive sequence based PCR fingerprinting following the manufacturer’s instructions (bioMérieux, Marcy-l’Etoile, France). The rep-PCR products were detected and analyzed using lab-on-a-chip microfluidics technology on an Agilent 2100 Bioanalyzer (Agilent, Santa Clara, CA, USA). Further analysis was performed with the web-based DiversiLab software (version 3.4) using the Pearson correlation coefficient to calculate pairwise similarities among all samples. In this study, we used 95% similarity thresholds for the data analysis (Voets et al., 2013).

### Whole Genome Sequencing and Analysis

A DNA library was prepared using the Nextera XT kit (Illumina, San Diego, CA, USA) according to the manufacturer’s instructions and then run on a Miseq (Illumina) for generating paired-end 250-bp reads. *De novo* assembly was performed using default settings of CLC Genomics Workbench v6.0.5 (CLC bio A/S, Denmark) as described previously (Ferdous et al., 2015). The MLST ST was also confirmed from the whole genome sequence by uploading the assembled genomes to the CGE MLST server (version 1.7; Larsen et al., 2012); the configuration for MLST was set to *E. coli* scheme 1. The resistome was generated by uploading assembled genomes to the CGE Resfinder server 2.0 (Zankari et al., 2012). The default settings for Resfinder were used except for the threshold of ID that was set to 85%. The serogenotype of the isolates was identified using CGE SerotypeFinder 1.0 (Joensen et al., 2015). The virulence genes were identified by mapping reads to a pseudomolecule generated by concatenating a set of *E. coli* virulence genes described before (Bielaszewska et al., 2011; Lima et al., 2013). In addition to the isolates of 2013 and 2011, the genomes of two isolates of 2009 (2009EL-2050, 2009EL-2071) were included for comparison of the virulence genes. The genome sequence of 2011C-3493 (GenBank accession no. NC_018658.1) was used as reference for extracting open reading frames (ORFs) from contigs of each strain by SeqSphere v1.0 (Ridom GmbH, Münster, Germany). Only the ORFs without premature stop codons and ambiguous nucleotides were extracted, and the sequences of those ORFs shared by all samples analyzed here were defined as the core genome for subsequent phylogenetic analysis (Leopold et al., 2014). According to the sequence identity of each ORF, a numerical allele type was assigned by SeqSphere. SeqSphere used the allelic profile formed by the combination of all alleles in each genome for constructing the minimum-spanning tree. This Whole Genome Shotgun project has been deposited at DDBJ/EMBL/GenBank under the accession number JRJF01000000 (*E. coli* 338), JRKD01000000 (*E. coli* 381-1), JRLM01000000 (*E. coli* 381-3), JRLD01000000 (*E. coli* 381-4), JRKE01000000 (*E. coli* 7N), JRLN00000000 (*E. coli* 8G), JRKF01000000 (*E. coli* LB227103). The version described in this paper is the first version.

## Results

### Demographic and Clinical Characteristics of Patients

Strain 338 was isolated from the fecal sample of a 22 year-old female patient (338) admitted to the Intensive Care Unit of the University Medical Center Groningen (UMCG) in July 2013, after returning from a holiday in Turkey. She presented with abdominal pain, bloody diarrhea, vomiting, and eventually developed HUS. Patient 381, a 24-year-old female traveling together with patient 338, showed symptoms of diarrhea, abdominal cramps, and abdominal pain but was not hospitalized.

### Primary Screening Results

Direct screening of feces by qPCR revealed the presence of *stx*1, *stx*2, and *escV* genes (patient 338) and *stx*2 gene (patient 381). Additional molecular screening of DNA isolated from enriched BGB broth for other virulence genes and O-serogroups resulted in detection of *aggR, aatA*, and *wzy_O104_* in both patients. Subsequently, as a result of colony screening for the presence of the *stx* gene and/or for ESBL production one *stx*2-positive non-ESBL-producing isolate (isolate 338) was obtained from patient 338, whereas two different *stx*2-positive isolates were recovered from patient 381: an ESBL producing isolate (381-1) and a non-ESBL producing isolate (381-4). In addition, one *stx*-negative but ESBL-producing isolate (381-3) was obtained from this patient. All *stx*-positive isolates contained the *aatA* and *terD* but not the *escV* gene, similar to the 2011 German outbreak isolates. No *stx*1-positive isolates were obtained. The *agg*R gene was detected in the 2013 and two of the three 2011 isolates (7N and 8G) but unexpectedly not in isolate LB227103. All *stx*-positive isolates were subtyped as *stx*2a and shared the same serotype (O104:H4) as the 2011 German outbreak isolates, whereas the *stx* negative ESBL producing isolate was serotype O126:H2. All isolates were sorbitol fermenting and tellurite resistant. MLST analysis showed that all 2013 EAEC Stx2a+ O104:H4 isolates belonged to ST678 as the 2011 outbreak strains, whereas the *stx-*negative isolate (381-3) was assigned to ST10. The screening results are summarized in **Table 1**.

### Phenotypic and Genotypic Resistance Profiles

Isolates 338 and 381-4 (both ESBL negative) were resistant to ampicillin and trimethoprim. Isolate 381-1 (ESBL positive) was resistant to ampicillin, cefotaxime, ceftazidime, cefepime, cefuroxime, and trimethoprim and this resistance pattern was almost identical to that of the *stx*-negative/ESBL positive isolate 381-3 from the same patient except that the latter was susceptible to trimethoprim. The resistance pattern of isolate 381-1 differed from that of three of the 2011 outbreak isolates which were resistant to amoxicillin-clavulanic acid, tetracycline and trimethoprim/sulfamethoxazole (**Table 2**).

**Table 2:** Phenotypic antibiotic resistance patterns and presence of corresponding antibiotic resistance genes among the isolates.

Isolates analyzed in this study (Phenotype/Presence of corresponding gene)							

	**2011 isolates**			**2013 isolates**			
		
**Name of Antibiotics**	**7N**	**8G**	**LB227103**	**338**	**381-1**	**381-4**	**381-3**
Ampicillin	R/*bla*_TEM-1_	R/*bla*_TEM-1_	R/*bla*_TEM-1_	R/*bla*_TEM-1_	R/*bla*_TEM-1_	R/*bla*_TEM-1_	R/*bla*_TEM-1_
Amoxicillin+ Clav acid	R/*bla*_TEM-1_	R/*bla*_TEM-1_	R/*bla*_TEM-1_	S	I	S	S
Cefuroxime	R/*bla*_CTX-M-15_	R/*bla*_CTX-M-15_	R/*bla*_CTX-M-15_	S/N	R/*bla*_CTX-M-15_	S/N	R/*bla*_CTX-M-15_
Cefotaxime	R/*bla_CTX-M-15_*	R/*bla_CTX-M-15_*	R/*bla_CTX-M-15_*	S/N	R/*bla_CTX-M-15_*	S/N	R/*bla_CTX-M-15_*
Ceftazidime	R/ *bla_CTX-M-15_*	R/ *bla_CTX-M-15_*	R/ *bla_CTX-M-15_*	S/N	R/ *bla_CTX-M-15_*	S/N	R/*bla_CTX-M-15_*
Cefepime	R/*bla_CTX-M-15_*	R/*bla_CTX-M-15_*	R/*bla_CTX-M-15_*	S/N	R/*bla_CTX-M-15_*	S/N	R/*bla_CTX-M-15_*
Co-trimoxazole	R/*sul1 & 2*	R/*sul1 & 2*	R/*sul1 & 2*	S/*sul1*^a^	S/s*ul1*^a^	S/*sul1*^a^	S/N
Tetracycline	R/*tetA*	R/*tetA*	R/*tetA*	S/N	S/N	S/N	S/N
Trimethoprime	R/*dfrA7*	R/*dfrA7*	R/*dfrA7*	R/*dfrA7*	R/*dfrA7*	R/*dfrA7*	S/N
Streptomycin	ND/*strA-B*	ND/*strA-B*	ND/*strA-B*	ND/N	ND/N	ND/N	ND/N


*(R, represents drug resistance; S, represents drug sensitivity; I, represents intermediate; N, represents not found; ND, represents not determined).*

*^a^*sul1* gene was deactivated by an *IS26* insertion.*

In agreement with the results of the phenotypic resistance pattern, the resistome of the three 2013 EAEC Stx2a+ O104:H4 isolates differed from that of the 2011 isolates: the *strA*, *strB* (streptomycin resistance), *sul2* (sulfonamide resistance), and *tetA* (tetracycline resistance) genes were only found in the 2011 outbreak isolates (**Table 2**). A *bla*_CTX-M-15_ gene was detected in isolate 381-1 (*stx*-positive) and 381-3 (*stx*-negative) as well as in the 2011 isolates. In addition, WGS showed the presence of the *dfrA7* gene in all isolates except 381-3 resulting in the latter’s trimethoprim-sensitive phenotype. A truncated *sul*1 gene resulting from an insertion of *IS26* was found in all EAEC Stx2a+ O104:H4 isolates.

### Virulence Profile

DNA microarray results showed that the virulence profile of the 2013 isolates was similar to that of the EAEC Stx2a+ O104:H4 isolates of 2011 and 2009 used in this study. The only difference was the presence of the microcin H47 biosynthesis gene *mchB* and *mchC*, which were identified in the 2009 and 2011 strains, but were absent in the EAEC Stx2a+ O104:H4 isolates of 2013.

The results obtained by WGS were identical to that of the DNA microarray and confirmed the absence of the *aggR* gene in the outbreak strain LB227103. WGS revealed the presence of several additional virulence genes including *aap*, *aaiC*, *aggA*, *lpfA_O113_, lpfA_O26_, fyuA*, *irp*2, and *set1* (not detected by PCR or microarray) in our isolates (**Table 3**).

**Table 3:** Virulence genes detected by qPCR, microarray and whole-genome sequencing (WGS) analysis.

Gene name	Function	7N	8G	LB227103	338	381-1	381-4	2009EL-2050	2009EL-2071	55989
*aap*^a^	Synthesis of anti-aggregation protein dispersin	+	+	+	+	+	+	+	+	+
*aatA*	Necessary for translocation of dispersin (Aap)	+	+	+	+	+	+	+	+	+
*aaiC*^a^	aggR-activated island C	+	+	+	+	+	+	+	+	+
*aggA*^a^ *(AAF/I)*	Pilin subunit of aggregative adherence fimbriae I	+	+	+	+	+	+	+	+	-
*aafA*^a^	Pilin subunit of aggregative adherence fimbriae II	-	-	-	-	-	-	-	-	-
*agg3A*^a^ *(AAF/III)*	Pilin subunit of aggregative adherence fimbriae III	-	-	-	-	-	-	-	-	+
*agg4A*^a^	Pilin subunit of aggregative adherence fimbriae IV	-	-	-	-	-	-	-	-	-
*Agg5A*^a^	Pilin subunit of aggregative adherence fimbriae V	-	-	-	-	-	-	-	-	-
*aggR*	Transcriptional regulator AggR	+	+	-	+	+	+	+	+	+
*astA (EAST1)*	heat-stable enterotoxin 1	-	-	-	-	-	-	-	-	+
*bfpA*	Bundle-forming pili	-	-	-	-	-	-	-	-	-
*cdt (I-V)*	Cytolethal distending toxin	-	-	-	-	-	-	-	-	-
*eae*	Intimin	-	-	-	-	-	-	-	-	-
*EHEC-hlyA*	EHEC haemolysin	-	-	-	-	-	-	-	-	-
*elt*	Heat-labile enterotoxin (LT)	-	-	-	-	-	-	-	-	-
*espP*	Serine protease	-	-	-	-	-	-	-	-	-
*est1a*	Heat-stable enterotoxin (STIa)	-	-	-	-	-	-	-	-	-
*est1b*	Heat-stable enterotoxin (STIb)	-	-	-	-	-	-	-	-	-
*fyuA*^a^	Component of iron uptake system on HPI	+	+	+	+	+	+	+	+	+
*ial*^a^	Invasive plasmid (pInv)	-	-	-	-	-	-	-	-	-
*iha*	Iron-regulated gene	+	+	+	+	+	+	+	+	+
*irp2*^a^	Component of iron uptake system on HPI	+	+	+	+	+	+	+	+	+
*lpfA_O113_*^a^	Structural subunit of LPF of STEC O113	+	+	+	+	+	+	+	+	+
*lpfA_O157-OI141_*^a^	Structural subunit of LPF of STEC O157:H7	-	-	-	-	-	-	-	-	-
*lpfA_O157-OI154_*^a^	Structural subunit of LPF of STEC O157:H7	-	-	-	-	-	-	-	-	-
*lpfA_O26_*^a^	Structural subunit of long polar fimbriae (LPF) of STEC O26	+	+	+	+	+	+	+	+	+
*mchB*	Synthesis of Microcin	+	+	+	-	-	-	+	+	-
*mchC*	Synthesis of Microcin	+	+	+	-	-	-	+	+	-
*mchF*	Synthesis of Microcin	+	+	+	+	+	+	+	+	-
*pet*	Plasmid-encoded toxin	-	-	-	-	-	-	-	-	-
*pic*	Gene encoding protein for intestinal colonization	+	+	+	+	+	+	+	+	+
*saa*	STEC autoagglutinating adhesin	-	-	-	-	-	-	-	-	-
*sat*	Secreted autotransporter toxin	-	-	-	-	-	-	-	-	-
*set1*^a^	Shigella enterotoxin 1	+	+	+	+	+	+	+	+	+
*sepA*	Shigella extracellular protease	+	+	+	+	+	+	+	+	-
*sfpA*	Plasmid-borne gene encoding the pilin subunit	-	-	-	-	-	-	-	-	-
*sigA*	Shigella IgA-like protease homolog	+	+	+	+	+	+	+	+	+
*stx1*	Shiga toxin 1	-	-	-	-	-	-	-	-	-
*stx2*	Shiga toxin 2	+	+	+	+	+	+	+	+	-
*subA*	Subtilase cytotoxin	-	-	-	-	-	-	-	-	-
*ter cluster*	Tellurite resistance	+	+	+	+	+	+	+	+	-


*^a^Data derived only from WGS.*

### Phylogenetic Analyses

**FIGURE 1:**
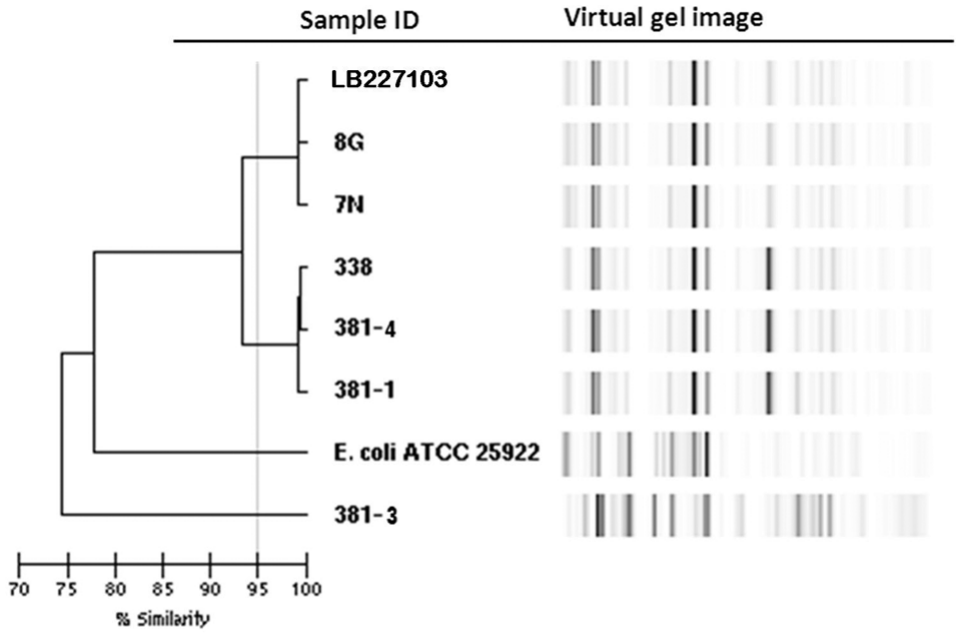
**Dendrogram of *Escherichia coli* isolates based on rep-PCR results.** Stx2a+ O104:H4 isolates of 2011 and 2013 are separated into two clusters with a similarity of less than 95% whereas the non O104:H4 *E. coli* isolates are clearly separated with a similarity of less than 80%.

**FIGURE 2:**
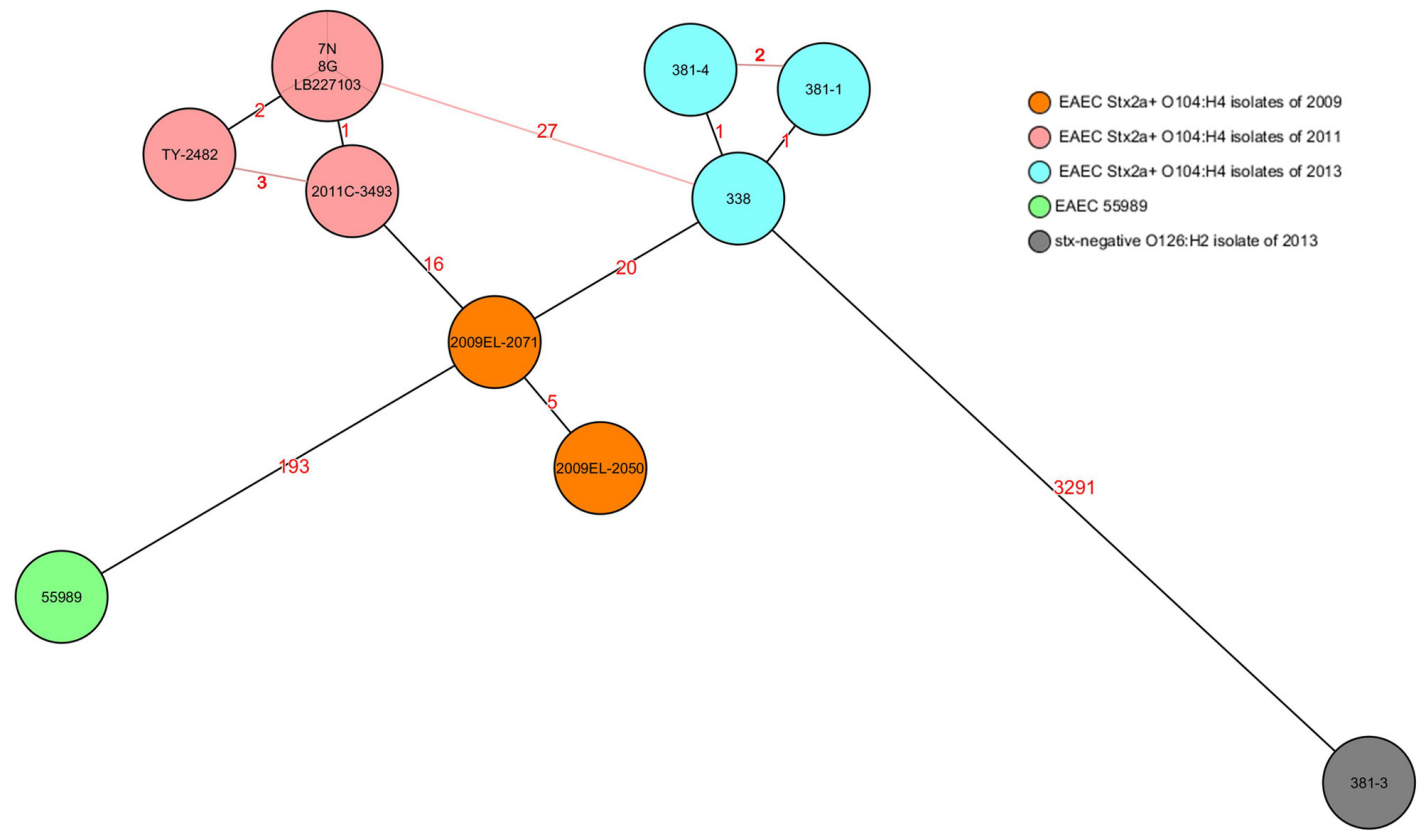
**Core-genome phylogenetic analysis of *E. coli* strains.** The minimum spanning tree was generated by SeqSphere and based on allelic profiles comparing 3764 alleles present in all analyzed strains and defined as their core genome. Numbers in the lines indicate the number of allele differences between isolates. Black lines indicate minimum distances and red lines connect isolates with more than minimum distances. Different colors represent isolates of different time periods except isolate 381-3, which was isolated in 2013 but marked in a different color as it is *stx* negative. EAEC 55989 was used as the hypothetical EAEC Stx2a+ O104:H4 progenitor.

Rep-PCR analysis was used to investigate the genetic relationship between the different isolates. EAEC Stx2a+ O104:H4 isolates of 2011 and 2013 clustered into two different groups sharing a similarity of less than 95% whereas the two non O104:H4 isolates (*E. coli* ATCC 25922 and *E. coli* O126:H2) separated from the O104:H4 clusters with less than 80% similarity (**Figure 1**). To type the different isolates with an even higher resolution, a core-genome phylogenetic analysis based on WGS results was performed. In total 3764 ORFs were shared by all isolates analyzed and these were defined as the core genome for further phylogenetic analysis in this study. The minimum-spanning tree shows that the three 2013 EAEC Stx2a+ O104:H4 isolates clustered together with one to two-allele difference among them. One-allele difference of isolate 381-1 from isolates 338 and 381-4 was found in the gene encoding a carboxyterminal protease and was caused by a non-synonymous point mutation (1481T > A) resulting in a premature protein (Leu494Stop) in isolate 381-1. In addition, one-allele difference of isolate 381-4 from isolates 338 and 381-1 was found in a gene encoding a putative cation:proton antiport protein caused by a non-synonymous point mutation (1465A > C) resulting in an amino-acid change in the protein (Ile489Leu) in isolate 381-4. No allele differences were found between the three 2011 outbreak isolates (7N, 8G, and LB227103), and they clustered together with two other reported outbreak strains (TY-2482 and 2011C-3493) with two to three-allele difference among them (**Figure 2**). The 2009 isolates (2009El-2071 and 2009El-2050) were separated from each other with a five-allele difference. The cluster of 2013 O104:H4 isolates were separated from the 2009 and 2011 O104:H4 clusters with a minimum of 20 and 27 different alleles, respectively (**Figure 2** and **Supplementary Table S1**). There were more than 193 different alleles between the typical EAEC strain 55989 (O104:H4 *stx*-negative) and the 2013 EAEC stx2a+ O104:H4 isolates, whereas isolate 381-3 (O126:H2, *stx*-negative) was far distinct from any of the O104:H4 strains analyzed, with more than 3291-allele differences (**Figure 2**).

## Discussion

In this study, EAEC Stx2a+ O104:H4 isolates obtained from stool samples of a HUS patient and her friend, traveling together to Turkey in 2013, were characterized and compared with the 2011 German EAEC Stx2a+ O104:H4 outbreak isolates and with two isolates from cases of bloody diarrhea that occurred in the Republic of Georgia in 2009. The phylogenetic relationship of these isolates was also compared with EAEC 55989, the hypothetical EAEC Stx2a+ O104:H4 progenitor strain isolated in central Africa in 1995. Besides using established molecular typing methods routinely used in our laboratory, WGS was used. Initially, the three EAEC Stx2a+ O104:H4 isolates obtained in 2013 were suspected to be similar to the 2011 German outbreak strains based on PCR results. The 2013 isolates could not be distinguished from the 2011 outbreak strains using a multiplex screening PCR targeting O104*wzy*, *fliCH4*, *stx2*, and *terD* genes as characteristic features of the 2011 outbreak strains (Bielaszewska et al., 2011; Zhang et al., 2012). Our microarray analyses revealed more detailed information but did not allow us to definitely discriminate the 2013 EAEC Stx2a+ O104:H4 isolates from the 2011 ones. Differences found between the 2011 and 2013 EAEC Stx2a+ O104:H4 isolates included two virulence genes (*mchB* and *mchC*) and three drug-resistant genes (*tetA, strA-B*, and *sul2*). However, these genes are known to be located in multiple genomic islands and could therefore be easily lost or obtained (Grad et al., 2013; Guy et al., 2013). Although rep-PCR separated the 2011 and 2013 EAEC Stx2a+ O104:H4 isolates into two clusters, this technique is not suitable for large-scale/global outbreak typing, as the inter-laboratory reproducibility is not sufficient for this. In addition, also the intra-laboratory reproducibility may vary making it necessary for an accurate typing to include all the isolates that need to be compared within the same run (Voets et al., 2013). The high resolution of WGS enabled us to discriminate the 2013 isolates from the 2011 German outbreak in the most accurate way.

In this study, a gene-by-gene typing approach also known as whole genome MLST (wgMLST), extended MLST, MLST+ or core genome MLST (cgMLST) was used (Maiden et al., 2013). The EAEC Stx2a+ O104:H4 isolates of 2013 appear to differ from those of 2011 and 2009 by a minimum of 27 and 20 alleles, respectively. This suggests that 2013 isolates were relatively closer related to the 2009 clone of southeast Europe than to the 2011 outbreak clone. Unfortunately, there is no established threshold for the MLST+ approach to address intra- and inter-cluster differences yet. In addition, only the core genome was used for constructing the phylogenetic tree minimizing the chance of including mobile genetic elements (MGEs) in the analysis as there is no consensus on how MGEs should be taken into account in bacterial phylogeny (Daubin et al., 2002; Frost et al., 2005). As the estimated mutation rate of *E. coli* is reported to be 1.1 nucleotide per genome per year (Reeves et al., 2011), the 27-allele difference between the 2013 and 2011 isolates suggests that the EAEC Stx2a+ O104:H4 isolates from the different time periods belong to different clones. Moreover, only a one-allele difference was found between the isolate of the HUS patient and that of her friend, strongly supporting the hypothesis that either both patients obtained STEC from the same source or that one transmitted STEC to the other.

In addition to high resolution typing, WGS provided us with more detailed molecular characteristics of the isolates. This helped us to explain in more detail the phenotypic results of the isolates. For example, an insertion within the *sul*1 gene resulting in protein truncation was found in all 2013 EAEC Stx2a+ O104:H4 isolates, which could explain why the isolates were susceptible to trimethoprim/sulfamethoxazole in spite of the presence of the *sul*1 gene detected by the microarray. On the other hand, a *sul*2 gene existing in the 2011 but not the 2013 isolates may confer resistance to trimethoprim/sulfamethoxazole in spite of the fact that also the 2011 isolates carried the truncated *sul*1.

Notably, one of the 2013 EAEC Stx2a+ O104:H4 isolates (381-1) was *bla*_CTX-M-15_ positive as the 2011 outbreak isolates. As isolate 381-3 (*stx*-negative/O126:H2/*bla*_CTX-M-15_) was also obtained from the same host, it is likely that 381-1 acquired this ESBL gene from isolate 381-3 or the other way around. Our study did, however, not address a detailed analysis of the MGEs and plasmids as the aim of the study was to show the value of WGS technique in routine clinical diagnostics to identify and compare potentially pathogenic strains.

Recently, two similar isolates of EAEC Stx2a+ O104:H4 causing bloody diarrhea and HUS were reported in Belgium. Patients of both cases traveled to Tunisia and Turkey, respectively, and the infection may have been acquired in those regions (De Rauw et al., 2014). This is similar to our and other studies describing EAEC Stx2a+ O104:H4 infections associated with traveling to Turkey, Tunisia, Egypt, and North Africa (Ecdc/Efsa, 2011; Grad et al., 2013).

Our study shows that WGS has the potential to be integrated in current routine laboratories to relatively rapidly obtain reproducible and detailed information in one single step. This facilitates hospitals and public health organizations to make appropriate strategies for infection control and public health measures in real time (Reuter et al., 2013). Surely, some challenges have to be overcome before applying WSG to routine diagnostics, like the availability of user-friendly bioinformatics tools, automation of the workflow and availability of genomic databases (Sherry et al., 2013).

## Conclusion

The detailed characterization of EAEC Stx2a+ O104:H4 isolates obtained in 2013 including the high resolution typing approach helped us to distinguish them from the 2011 German outbreak strains. It further supported the general idea that, although the 2011 outbreak is stopped, EAEC Stx2a+ O104:H4 strains highly similar to the 2011 outbreak strain in their core genome still circulate. They still pose a serious risk for public health being a potential source for causing a new EAEC Stx2a+ O104:H4 outbreak if not monitored carefully.

## Author Contributions

Study design: MF, KZ, JR, AF

Performing Experiments: MF, KZ, RdB

Acquisition of data: MF, KZ, RdB

Analysis and interpretation of data: MF, KZ, AK-S, JR

Drafting of manuscript: MF, KZ, JR, AF

Critical revision: MF, KZ, RdB, AK-S, JR, AF

## Conflict of Interest Statement

The authors declare that the research was conducted in the absence of any commercial or financial relationships that could be construed as a potential conflict of interest.
